# A Case of Cuprum Poisoning

**Published:** 1893-05

**Authors:** John McE. Ward

**Affiliations:** Philadelphia


### A CASE OF CUPRUM POISONING.

JOHN M'E. WARD, M. D., PHILADELPHIA.

Female, æt. twenty-four, brunette, rather tall, well developed, intelligent, sanguine temperament, single.

Previous and family history good.

Has had measles and whooping cough before the ninth year. No diseases since. First menstruated at thirteen years, dysmenorrhœa being a constant attendant. Bowels and urine previously normal.

Five years ago accepted a situation and has since been continuously employed in a factory weaving the insulation on copper wire, used for electrical purposes. Minute particles of the metal, with dust, etc., are continuously floating about, in quantity sufficient to require sweeping the floor twice daily.

Presented herself to me September 20th, 1892, and was under observation and treatment until January 23d, 1893.

*History:* In early part of July, while working in the mill, without any warning whatever, fell in a "fit," accompanied by an intense griping abdominal pain; was unconscious, frothed at the mouth, and lost both urine and feces involuntarily. The spasm lasted from three to five minutes, and was so violent as to require several attendants to "hold her down." With returning consciousness came the abdominal pain, which was severe enough to bring on another convulsion.

A doctor (allopath) was sent for, who diagnosed "menstrual colic," and after having her removed home, prescribed "something which made her sleep."

She remained in bed one week and was under his care two weeks, the "fits" continuing, one to five in twenty-four hours; also the abdominal pains, etc. At this point was transferred to my care.

*Status presens:* Intensely nervous, is in *constant motion*, hands picking at clothing, handkerchief, etc.; *feet moving incessantly*, whether sitting or standing.

On walking, *staggers* as if intoxicated, can't control legs so as to walk a straight seam in carpet.

On standing or walking, the *knees "give out"* and she "falls in a heap," this occurs repeatedly.

The abdominal pain is severe, and seems to *concentrate from all directions to a point at the middle of tranverse colon, it never radiates, always concentrates* (as do the spokes of a wheel to the hub) *at which time it feels as if a lump were there* (intussusception).

The pains are worse from breathing, walking, or motion of any kind. Better when sitting doubled up, but not relieved by pressure.

On looking up suddenly, dizziness occurs, sensation as if looking through a veil, sometimes momentary obscuration of sight, *followed by vertigo*, would fall unless help were at hand. Eyes feel bruised and have dark circles around them. Headache in forehead and between the eyes. *Slow and fairly regular movement of eyeballs from side to side.*

*Insomnia, restlessness, wants to go somewhere or do something all the time, but movement seems to make all symptoms worse.*

Cannot control her mind so as to do anything.

Frequent daily nausea and *retching*. Better when lying down, *fasting;* would go several days without food on this account.

All stomach symptoms better from *warm* milk. Stomach and abdomen considerably swollen, cannot make clothes meet.

Taste decidedly coppery; no taste for foods, everything tastes alike; fluids choke her; tongue coated light brown in centre; dry mouth but no thirst.

*Persistent, painless diarrhœa, not exhausting,* however; *feels better after a passage.*

*There is involuntary defecation and urination during a convulsion and a free, clear, voluntary urination, with great relief, afterward. Coryza and epistaxis almost always follow a convulsion.*

Intense soreness of entire muscular system; temporary paralysis of right side, *leg mostly affected.* (This wears away in fifteen to thirty minutes.)

The menses have been totally suppressed for five months, there is leucorrhœa with pruritis; vagina usually hot and dry.

*Treatment:* The history of the case and symptoms led me to Copper as the cause of the trouble. I therefore gave Hepar$^{12}$, as being antidotal, four doses daily; this was continued for five weeks, with most excellent results, nearly all the prominent symptoms clearing up. One dose of Pulsatilla served to re-establish the menstrual function in three days, and that without the dysmenorrhœa that had formerly existed. The nervous and gastric disturbances were successfully treated with Nux-vom.

From this time to the end of treatment, she has been receiving Hepar$^{30}$ and an occasional dose of Ignatia and Dulcamara as symptoms required.

Since January 23d—four months after beginning treatment—she has been free of all symptoms; nervous troubles have disappeared; appetite good; menstruates regularly and without discomfort. She is now engaged in another business.

It is also worthy of remark that several other women employed in the same work have been similarly affected, but in a modified degree, and in *none* of the cases, so far as I can ascertain, was there a diagnosis of "copper poisoning."

